# Copolyamide–Clay Nanotube Polymer Composite Nanofiber Membranes: Preparation, Characterization and Its Asymmetric Wettability Driven Oil/Water Emulsion Separation towards Sewage Remediation

**DOI:** 10.3390/polym13213710

**Published:** 2021-10-27

**Authors:** Sneha Bhagyaraj, Patrik Sobolčiak, Mohammad A. Al-Ghouti, Igor Krupa

**Affiliations:** 1 Center for Advanced Materials, Qatar University, Doha P.O. Box 2713, Qatar; snehamkoottungal@gmail.com (S.B.); patrik@qu.edu.qa (P.S.); 2 Department of Biological and Environmental Sciences, College of Arts and Sciences, Qatar University, Doha P.O. Box 2713, Qatar; mohammad.alghouti@qu.edu.qa

**Keywords:** halloysite, copolyamide, membrane, emulsion separation, contact angle

## Abstract

To address the problem of ever-increasing oily wastewater management, due to its directional liquid transport property, membranes with asymmetric wettability can be effectively used for emulsion separation. This study reports the synthesis of electrospun polymer–clay nanocomposite nanofibers, using co-polyamide polymer (COPA) and halloysite nanotubes (HA) as filler. The influence of clay content on the morphological, thermal and dielectric properties of the polymer composite nanofiber was investigated comprehensively to address the material characteristics of the developed system. The surface structure analysis and contact angle measurements of the electrospun composite nanofibers confirms the change in surface roughness and wettability when the fillers are added to the polymer. The porosity of the composite electrospun nanofiber membrane was found to be 85% with an oil adsorption capacity of 97% and water permeability of 6265 L/m^2^ h. Furthermore, the asymmetric wettability-driven oil/water emulsion separation abilities of the as-synthesized membranes shows that the separation efficiency of the composite fiber membrane is 10% improved compared to that of the neat fiber membrane, with improved separation time.

## 1. Introduction

A major threat to the natural balance of the ecological environment, especially the aquatic ecosystem, is the oil pollution caused by the food, petrochemical and textile industries [1,2]. With the fast and enormous development of food-related industries, including catering and restaurants, the release of untreated sewage waste water into various water sources is also at its peak. The immoderate discharge of untreated waste water containing harmful pollutants, such as insoluble oils and organic contents into different water bodies, can wreak havoc on the marine ecosystem. Oil/water mixtures are classified into various categories based on the droplet size, and among them, emulsions with droplets size ≤20 µm are known to be difficult to separate [3]. In particular, oil/water emulsions under 20 µm in size cause a serious problem in water treatment. The limitations of conventional techniques employed in sewage waste water treatments, such as high energy cost and low separation efficiency have raised the interest of researchers to find more advanced separation mechanisms specially for oil/water emulsions.

Separation science and technology, especially water treatment, have gained promising advancement with the introduction of polymers and polymer nanocomposites [4,5,6]. From the wide variety of polymers available, the real challenge is to identify the proper candidate suitable for the respective application. Various factors, such as molecular weight, the presence of functional groups, surface charge, etc., play a crucial role in determining the properties of polymers [7,8]. Compared to conventional methods, membrane technology is highlighted as one of the best methods for the separation of oil from oil/water mixtures considering its feasibility, environmental friendliness, high productivity, small footprint and cost effectiveness [9,10,11].

Among various kinds of membranes, electrospun polymeric nanofiber membranes have been widely studied as suitable membranes for the purpose of oil/water separation applications [12]. By electrospinning techniques, nanofibers with a controllable structure, high surface-to-volume ratio, interconnected pores, high porosity and controllable pore size distribution and composition can be prepared [13]. Various properties of the fiber mats, including size, porosity and surface energy, can be tailored by varying different parameters, such as the polymer solution concentration, applied voltage, and flow rate [14]. However, the polar polymer-based membranes encounter some disadvantages, due to their tendency to adsorb the organic pollutants, such as the blockage of pores, thereby decreasing the flux and separation efficiency in long time runs [15]. Recently, to overcome the above problems in practical application of nanospun membranes, researchers have focused on tailoring the wettability of the membrane surfaces by combining various functional materials for the efficient separation of oil/water emulsions [16]. Zhang et al. fabricated a s-kaolin particles–modified PAN composite membrane with super hydrophobic and superoleophilic properties through electrospinning [17]. The PAN/s-kaolin composite membrane exhibited an excellent oil adsorption capability with around 37.2 g/g capacity.

Asymmetric wettability membranes (AWM) allow the fast directional transport of water and can play an important role in oil/water separation [18]. On hydrophobic surfaces, water tends to quickly penetrate and move to the hydrophilic area; however, it cannot be transferred to the opposite side unless an external pressure is applied. For porous substrates, other than the surface structure, the capillary effect also plays an important role in liquid transport. As per the Young–Laplace equation, the capillary pressure (Pc) of a liquid in a cylindrical pore (radius, r) depends on the contact angle (θ) and the surface tension (γ) of the liquid. When θ < 90°, the capillary pressure is high and the liquid goes into the pore and when θ > 90°, the pore resists the flow of liquid.

The membranes used in the case of energy-intensive cross-flow filtration of oil/water separation are hydrophobic in nature [19]. Developing super hydrophobic membranes and membranes with asymmetric wettability is a promising area to explore for the oil/water separation application [20]. Typically, asymmetric wettability membranes are developed by methods such as coating and functionalization of the membrane surface, using various nanoparticles [21,22]. These methods are sometimes complicated and also have the disadvantage of causing the leakage of nanoparticles on water, affecting the permeation rate, due to surface coating and degradation of the membrane [23]. Preparing a membrane using a polymer filler composite structure with adequate surface properties and asymmetric wettability can help to overcome some of these mentioned disadvantages. Li et al. reported nanofibrous membrane with a hierarchical caterpillar-like structure by growing nickel–cobalt layered double hydroxides on PAN (polyacrylonitrile) electrospun nanofibers, which improved the superhydrophilicity, underwater superoleophobicity and enhanced oil-repellency performance [24].

Halloysite clay nanotubes are layered aluminosilicates (Al_2_Si_2_O_5_(OH)_4_·nH_2_O) (HA), with a large surface area and active pore sites, which are extensively used for numerous applications, including catalyst, polymer reinforcement, drug delivery, pollutants removal, and thermal energy storage [25,26,27]. Natural HA are sensitive to moisture and hydrophilic because of gibbsite octahedral sheet (Al–OH) groups existing on the internal surface. Song et al. reported modified cotton fabrics, using octadecyl trimethoxy siloxane modified halloysite nanotubes with a superhydrophobic surface for oil water separation with 99.99% efficiency [28]. However, there is always a possibility of the leakage of halloysite nanotubes from the cotton surface. Wang et al. reported modified halloysite nanotubes anchored at the polyvinylidene fluoride/graphene oxide membrane surface via polydopamine adhesive, which exhibited a pure water flux of 1500 L m^−2^ h^−1^ under gravity and a separation efficiency of up to 99.5% for oil/water emulsion separation [29].

From previous research studies, it is evident that combining the properties of nanoparticles with a large porosity, surface area and membrane can effectively lead to the development of novel multifunctional material for the purification of waste water [30]. The efficiency of a composite membrane depends on the effective interaction of the inorganic nanoparticle and the organic polymer. Therefore, exploring a suitable method for the stable loading of nanoparticles in the composite structure is extremely important.

Based on the above elaborations, for the first time, we report the synthesis of copolyamide–halloysite clay nanotube polymer nanocomposite nanofiber membranes, using n-isopropanol as the solvent. The as-synthesized membrane showed asymmetric wettability, due to its surface roughness and other surface characteristics. It is noteworthy that in this system, the possibility of secondary pollution, due to the leakage of the nanoparticle, is highly reduced. Detailed chemical, morphological, thermal and electrical characterization of the as-prepared composite membrane was performed. Changes in its surface wettability were examined in detail, using contact angle measurements followed by a study of its oil/water separation efficiency.

## 2. Materials and Methods

### 2.1. Materials

Co–polyamide (Vestamelt X1010, EVONIK Industries, Essen, Germany), halloysite nanoclay (HA, kaolin clay, obtained from Sigma Aldrich, Shanghai, China), n-propanol > 99.5% (Sigma Aldrich, St. Louis, MO, USA), corn oil (0.917 g/cm^3^) (Cornlite, Nashik, India), were all used in this experiment. Ultra-pure water (prepared by Purification System Direct Q3, Millipore Corporation, Molsheim, France), formamide > 99.5% (Sigma Aldrich, St. Louis, MO, USA), and ethylene glycol >99% (Sigma Aldrich, St. Louis, MO, USA) were used as testing liquids for the contact angle measurements.

### 2.2. Preparation of Copolyamide/HA(COHA) Nanocomposite Fiber

Initially, 15 wt% solution of the copolyamide polymer was prepared by dissolving Vestamelt X 100 in n-propanol at 80 °C with continuous vigorous stirring for 3 h. The solution was then stirred overnight at room temperature to make it uniform. For preparing the copolyamide–nanoclay composite solution (COHA), first, an appropriate amount of HA was dispersed in n-propanol, using probe sonication for 30 min (amplitude 70%, impulse 0.5) followed by the addition of Vestamelt X 100. The solution was then stirred at 80 °C in an oil bath for 3 h followed by overnight stirring at room temperature. Finally, the solution was sonicated, using a bath sonicator for 10 min to remove the trapped air bubbles. Different solutions were prepared by varying the concentration of HA from 0 wt% to 1.5 wt% and by keeping all the other procedures the same.

Nanofibers were fabricated using a NanoBond (Shenzhen, China) electrospinning device. Initially, 5 mL of copolyamide-HA solution (COHA) was taken in a 10 mL syringe with a stainless-steel type 8 needle. The tip of the needle was connected to a DC voltage supply in the range 0–50 kV. A rotating drum connected to the other electrode was covered with aluminum foil to act as the collector, which was kept at a distance of 10 cm from the tip of the needle. The electrospinning was carried out at room temperature at a voltage of 18 kV, with a flowrate of 0.5 mL/h and drum speed of 250 RPM. The process was continued for 2 h in order to obtain sufficient thickness of the membrane, which was approximately 65 µm.

### 2.3. Transmission Electron Microscopy (TEM)

Transmission electron microscopy (TEM) images of the HA and the composite nanofiber COHA were done, using a JEOL JEM-3010 electron microscope (Tokyo, Japan) operating at 200 kV. The clay sample was dispersed well in ethanol and a drop was casted on the TEM grid followed by drying. For the TEM image of the composite nanofiber, the COHA solution was spin coated on a copper grid for 5 s and then dried and viewed under the microscope.

### 2.4. Scanning Electron Microscopy (SEM)

The surface morphology of the electrospun membranes was examined with field emission scanning electron microscopy (FE-SEM, Nova Nano SEM 650, Hitachi, Tokyo, Japan) equipped with an energy-dispersive X-ray spectrometer (EDS). All specimens were sputter coated with 2 nm of gold before the SEM images were taken. The average fiber diameter and pore size of the electrospun fibers were measured from the SEM images, using the ImageJ software by considering 100 random locations [31]. The thickness of the membrane was measured, using a micrometer. The energy-dispersive spectroscopy (EDS) measurements were performed from three points of the specimens to understand the elemental composition of the samples.

### 2.5. Surface Analysis Using Optical Microscopy

The surface roughness of the samples was analyzed, using an Optical Surface Metrology System Leica DCM8, which uses non-destructive confocal technology to examine the steep inclinations up to 70 percent on the sample surface. The 3D images of the membranes were obtained, using an EPI 100× -L objective. The surface roughness changes were evaluated using the Sa parameter, which expresses an absolute height difference value of each point, compared to the arithmetic mean of the surface represented by Equation (1).

(1) $${\mathrm{Sa}}_{\;}=\iint_{a}\left|Z\;\left(x,y\right)\right|/dxdy$$

where *a* implies that the integration is performed over the area and then normalized by the cross-sectional area.

### 2.6. Fourier Transformed Infrared Spectroscopy (FTIR)

Fourier transformed infrared spectroscopy with attenuated total reflectance (FTIR-ATR) was performed to study the effective functional groups present in the composite fiber membrane using a Spectrum 400 (Perkin Elmer, Waltham, MA, USA).

### 2.7. X-ray Diffraction (XRD) Analysis

X-ray diffraction (XRD) analysis was performed on a Bruker D8 ADVANCE X-ray diffractometer (Bruker Corp., Billerica, MA, USA) equipped with Cu Kα radiation (λ = 0.154 nm) along a scanning range (2θ) 5° to 60° at a scan speed of 2°/min.

### 2.8. Thermogravimetric Analysis (TGA)

Thermal characterizations of the membranes performed done using thermogravimetric analysis (TGA, 4000, Perkin Elmer, Greenville, SC, USA) in the temperature range from 30 °C to 800 °C at a heating rate of 10 °C/min under a nitrogen atmosphere. The flow rate of nitrogen gas through the instrument during the reaction was 20 mL × min^−1^. For each measurement, approximately 15 mg samples were used.

### 2.9. Broadband Dielectric Spectroscopy (BDS)

To understand the molecular dynamics of the polymer systems under consideration, dielectric spectroscopy was carried out by monitoring the relaxation processes under an electric field. On the application level, dielectric property is very significant in the sense that they represent the capability to either store or dissipate energy by that particular material. Broadband dielectric spectroscopy (BDS) measurements were performed using a Novocontrol Concept 40 instrument with an Alpha dielectric spectrometer provided by Novocontrol Technologies GmbH (Montabaur, Westerwaldkreis, Germany). The measurements were carried out at room temperature in the frequency range of 10^3^–10^7^ Hz.

### 2.10. UV-Visible Spectroscopy

UV-visible spectra of the emulsions and permeates for the oil/water emulsion separation experiments were recorded with a spectrometer (SEC2000-UV/VIS, ALS, Tokyo, Japan), using a quartz cuvette.

### 2.11. Contact Angle Analysis

The surface contact angle measurements of the as-prepared composite nanofibers were done by an OCA35 optical system (Data Physics, Filderstadt, Germany). The method used was a sessile drop technique in which a volume of 2 µL of each testing liquid was used to measure the wettability of the prepared samples. Different solvents, such as distilled water, formamide, ethylene glycol and vegetable oil with different surface tensions were used. The angle between the interfaces (solid/liquid and liquid/vapor) is referred to as the contact angle. Six separate readings were taken for each sample and an average of the same was considered final.

### 2.12. Optical Microscopy

An inverted optical microscope (AxioCAM ERc 5s, ZEISS, Gottingen, Germany) was used for obtaining images of emulsions and permeates after the emulsion separation experiments, using the as-prepared membranes.

### 2.13. Porosity Calculation

The membrane porosities were evaluated by comparing the densities of the electrospun membrane and the bulk material, using the following equation:

(2) $$\mathrm{Porosity}\;(\%)=(1-\;\frac{\rho_{e}}{\rho_{b}})\;\times \;100$$

where *ρ_e_* density of the electrospun material and *ρ_b_* is the density of the bulk material.

The densities of the materials were calculated based on the dimensions and the weight of the specimens. Casted membranes of the same composition of the electrospun membranes were prepared, and the densities of both were compared. Gravimetric methods were used to measure the mass; meanwhile, the volume was calculated from sample dimensions. Finally, the equation was used to calculate porosity from measured densities.

### 2.14. Oil-in-Water Emulsion Separation Experiment

For the oil–water emulsion separation experiment, initially different concentrations of oil/water emulsions were prepared by varying the concentration of oil from 100 to 1000 ppm and adding sodium dodecyl sulfate (SDS) as a surfactant. The oil/water mixture was probe sonicated for 30 min to ensure good dispersion followed by vigorous shaking for one hour. For the separation experiment, the membranes were fixed between the intersection of a glass filter funnel and a vacuum filter flask. The effective separation area of the membrane depends on the diameter of the glass funnel used, which, in this case, was 9.58 cm^2^. A total of 250 mL of the oil/water emulsion was poured slowly into the top of the membrane, and the filtration was achieved through the vacuum-assisted method. The efficiency of separation was measured by analyzing the filtrate, using the UV visible spectroscopy and total organic carbon (TOC, automatic TOC-L, Shimadzu Corporation, Kyoto, Japan) measurements using the following equation:

(3) $$S_{eff}=100-\frac{c_{f}}{c_{s}}\times 100$$

where *S_eff_* is the efficiency of separation in %, *c_f_* is the concentration of oil in the filtrate in ppm, and *c_s_* is the concentration of oil in the stock solution in ppm.

Calibration fits were used to obtain the concentration of oil in water for UV spectroscopy. The oil content remaining in the filtrate was determined from the UV calibration curve (at a wavelength of 400 nm). The flux (J) through the membrane was calculated using the following equation:

(4) $$J=\frac{V}{At}\left(L/m^{2}h\right)$$

where *J* is the flux, *V* is the permeate volume (L), *A* is the effective test area (m^2^) and *t* is the time (h).

The oil adsorbed on the electrospun membranes were extracted, using n-hexane, and the test was performed several times to test the reusability of the membrane.

## 3. Results and Discussion

### 3.1. Characterizations of Copolyamide-HA Nanocomposite Nanofiber Membranes

#### 3.1.1. Scanning Electron Microscope (SEM) Analysis

**Figure 1:**
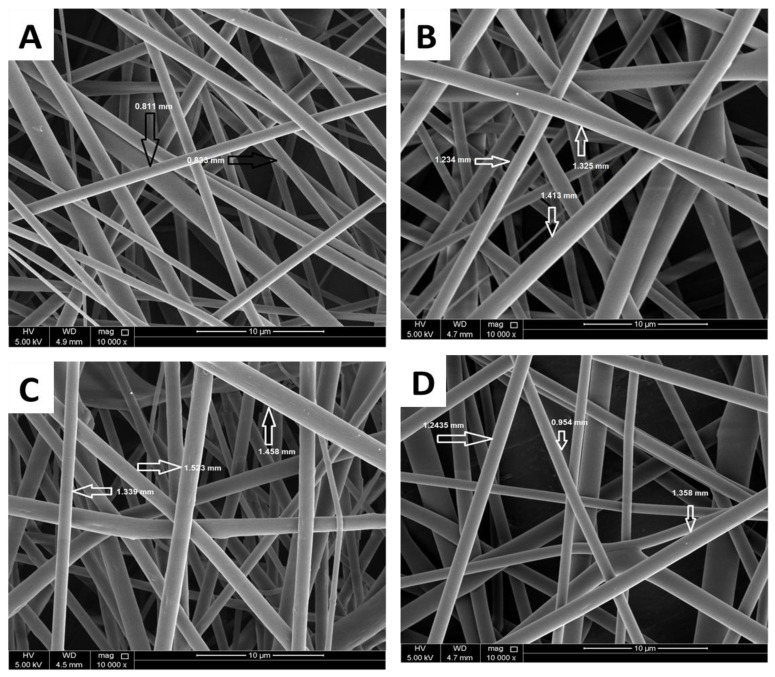
SEM images of copolyamide-HA composite nanofibers (**A**) 0% HA (**B**) 0.5% HA (**C**) 1% HA, and (**D**) 1.5% HA.

Figure 1 represents the SEM images of the COHA composite nanofiber containing various concentrations of the halloysite nanotube clay. The SEM images revealed a smooth and uniform fiber structure for all the compositions. The average diameters of the electrospun fibers measured from SEM were 966 ± 200 nm, 1265 ± 152 nm, 1349 ± 250 nm and 1040 ± 145 nm for COPA, COHA_0.5_, COHA_1_ and COHA_1.5_, respectively. One among the factors affecting the fiber diameter is the viscosity of the electrospinning solution. In this study, the viscosity of various solutions was found to be between 1.41 Pas and 1.84 Pas (Table 1). There was no drastic difference between the viscosity of the polymer solution and the polymer–filler solution; hence the fiber diameter difference is also not too high. Moreover, a small quantity of nanofillers does not affect the viscosity of the solutions drastically, thereby justifying the results. The average pore size of the membrane estimated was found to be 701 ± 110 nm, 756 ± 116 nm, 810 ± 212 nm and 840 ± 227 nm for COPA, COHA_0.5_, COHA_1_ and COHA_1.5_, respectively.

#### 3.1.2. Transmission Electron Microscopy (TEM) Analysis

**Figure 2:**
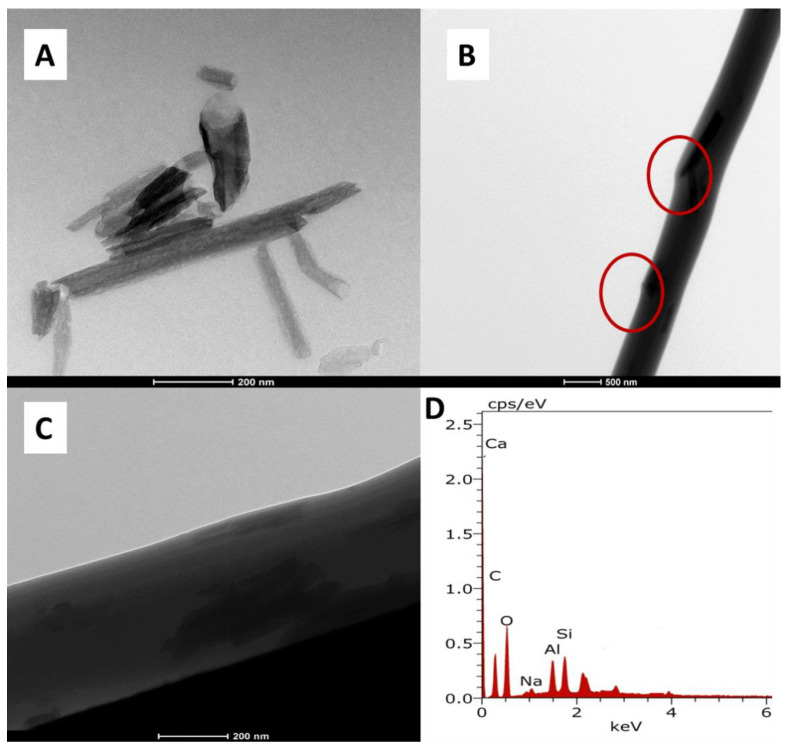
Transmission electron microscope images of (**A**) Halloysite nanoclay (**B**,**C**) copolyamide–HA composite nanofiber at different magnifications, (**D**) EDX spectra of COHA_1.5_.

Figure 2A represents the transmission electron microscope image of the halloysite clay nanotube. The tubular nature of the nanoclay is clearly revealed from the image, indicating a cylindrical shape and open-ended lumen. The average outer diameter for the nanoclay was found to be 45–90 nm with an inner lumen diameter of 10–20 nm with a corresponding wall thickness of around 25 nm. To confirm the presence and dispersion of halloysite inside the polymer fiber, a transmission electron microscope image of the single composite fiber was taken. From Figure 2B,C, it is clear that tubular shaped halloysite nanoclay in a well-dispersed form is present inside the polymer electrospun fiber. From the TEM image, it is visible that the tubular structure of the clay causes some projection, such as uneven formations on the surface of fiber, which might also contribute to the resulting surface roughness of the composite fiber. The addition of HNTs causes an increase in surface roughness, which was confirmed from the profilometer study results (Section 3.2). Tthe presence of halloysite nanotubes in the fiber is again confirmed from the electron dispersive spectrum (EDX) data, which is shown in Figure 2D.

#### 3.1.3. Profilometry Analysis

**Figure 3:**
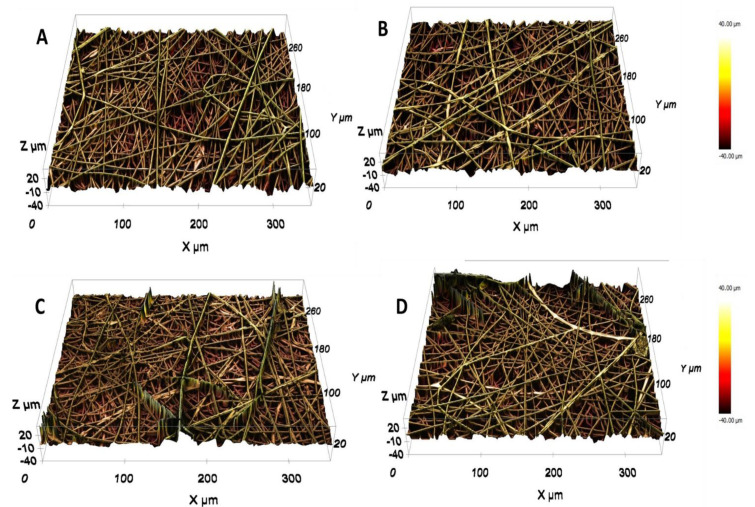
The 3D images of prepared samples obtained by the 3D optical surface metrology system: (**A**) COPA (Sa = 3.4 μm), (**B**) COHA_0.5_ (Sa = 4.5 μm), (**C**) COHA_1_ (Sa = 5.8 μm), (**D**) COHA_1.5_ (Sa = 6.6 μm); Sa represents the roughness parameter.

The 3D surface morphology of the prepared fibers was analyzed, using an optical surface metrology system; the images are shown in Figure 3. The non-destructive confocal scanning images also give information on the surface roughness represented by Sa (arithmetic mean height). The minimum Sa value was found to be 3.4 µm for the neat COPA, while the COHA_1.5_ showed a Sa value of 6.6 µm. From the 3D images, it is again clear that the fibers were homogenous with no beads and exhibited a regular appearance.

#### 3.1.4. Fourier Infrared Spectroscopy (FTIR) Analysis

**Figure 4:**
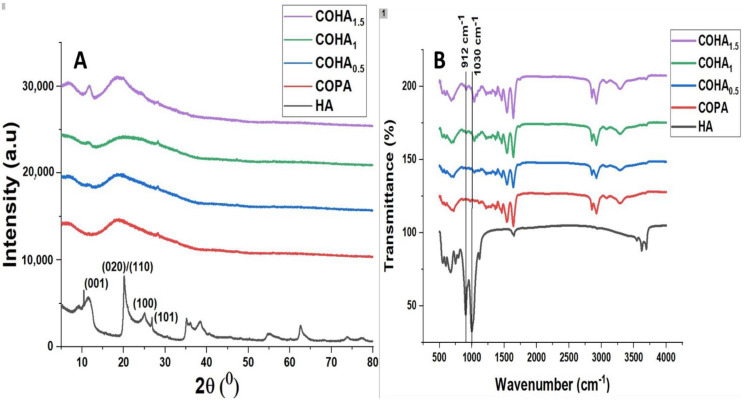
(**A**) FTIR spectra for copolyamide–halloysite composite nanofibers (**B**) XRD patterns for copolyamide–halloysite clay nanocomposite nanofibers.

Figure 4A represents the FTIR spectra of the as-synthesized COHA composite nanofibers. In the case of copolyamide, the peak at 3288 cm^−1^ represents the N–H stretching vibration. Two peaks at 2923 cm^−1^ and 2854 cm^−1^ represent the asymmetric –CH_2_ stretching and symmetric –CH_2_ stretching, respectively [32]. The band at 1637 cm^−1^ represents the –C=O stretching vibration. A sharp band at 1549 cm^−1^ represents the –NH bending vibration. The absorption bands at 3695 cm^−1^ and 3620 cm^−1^ present in the halloysite clay were assigned to the stretching vibration of the -OH groups at the inner surface of halloysite [33]. These bands were not prominently seen in the composite, which may be due to the fact that the surface of the HA is completely covered by the polymer. Two prominent bands present in the HA, 912 cm^−1^, which is due to the vibrations of the inner surface hydroxyl group and 1030 cm^−1^ due to the stretching vibrations of Si–O–Si, was not significant when the percentage of HA was small in the composite membrane. As the amount of HA increases, these peaks begin to appear in the spectrum, thereby confirming the presence of HA inside the composite membrane.

#### 3.1.5. X-ray Diffraction Analysis

Figure 4B represents the X-ray diffraction pattern of the copolyamidehalloysite nanoclay composite membranes. The X-ray diffraction pattern of the halloysite nanoclay, is fully consistent with the halloysite of basal spacing 7.4 A° (ICDD file no 29–1487). Two sharp peaks at 24.32° corresponding to (100) refection (ICDD file no. 33–1161) due to quartz impurity and 25.62° corresponding to (101) reflection of cristobalite (ICDD file no. 39–1425) were seen in the spectrum of halloysite clay [34,35]. The reflections at 12° (001) correspond to a basal spacing of 0.74 nm and at 20.4 (020)/(110) correspond to 0.44 [36]. Copolyamide, being a semi-crystalline polymer, did not show any significant peaks in the spectrum. After the addition of HA into the polymer matrix, the characteristic peak of halloysite clay at 12° due to the basal spacing of 0.74 nm appeared in the XRD spectrum of the composite. This confirmed the presence of halloysite clay inside the membrane.

#### 3.1.6. Thermogravimetric Analysis (TGA)

**Figure 5:**
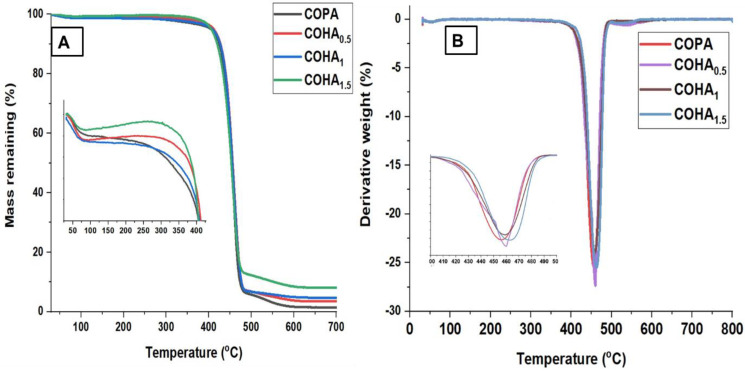
(**A**) TGA and (**B**) DTA curves of neat copolymer and COHA polymer nanocomposites.

The effect of HA on the thermal stability of the nanofiber membrane was studied, using thermogravimetric analysis. In this study, the weight decreasing pattern with respect to temperature is represented by the TGA curve, and the maximum temperature needed for the complete thermal degradation is represented by differential thermal analysis (DTA) curves. Figure 5A,B represents the TGA and DTA curves of the COHA polymer nanocomposite, respectively.

The TGA curve of halloysite clay shows mainly two stages of degradation. An initial endothermic peak around 55 °C, which shows loss of adsorbed water, and an endothermic peak around 502 °C due to dehydroxylation of the Al–OH groups [37]. The TGA curve for neat COPA clearly shows that the degradation takes place in two steps. The first stage is mainly due to the degradation of the aliphatic segments and the second stage due to the aromatic moieties present. The COPA was stable up to 400 °C and COHA was stable up to 423 °C, which indicates the highest processing temperature that can be adopted in this system. The decomposition of neat COPA occurs in the range of 406 °C–484 °C with one DTG peak at 455 °C. The thermal degradation of COHA_1.5_ occurs in the range of 422 °C–493 °C with one DTG peak at 462 °C. The ash content increase for the composite was due to the increased mineral content from the halloysite clay. Introducing a small quantity of halloysite clay into the copolymer resulted in the improvement of the temperature degradation profile of the composite, with the temperature values shifting to higher region.

#### 3.1.7. Dielectric Spectroscopy Analysis

**Figure 6:**
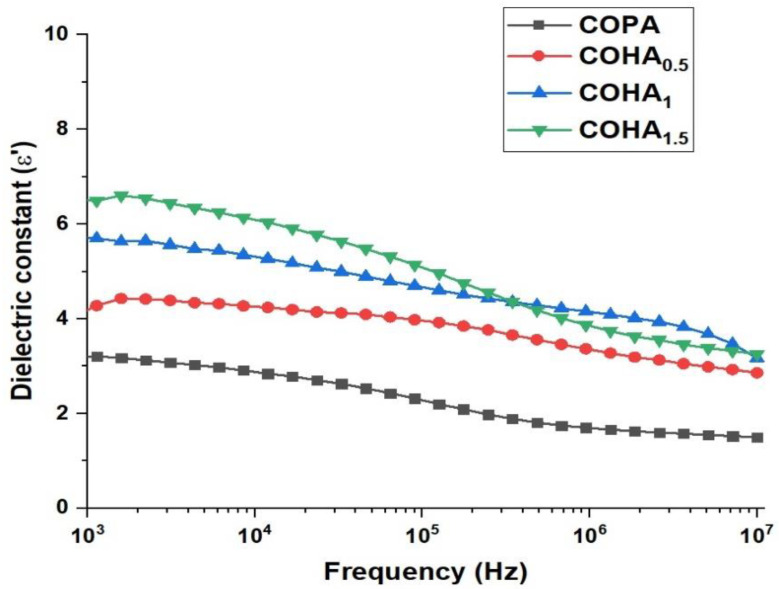
Frequency dependence of real permittivity (E’) for neat COPA and COHA nanocomposite fibers.

The frequency-dependent dielectric constant of the as-prepared neat COPA and COHA composite nanofibers with various filler content are studied over a frequency range of 10^2^ to 10^6^ and the results are shown in Figure 6. With the addition of HA to the neat COPA, the dielectric constant of the composite increases as expected [38]. This can be attributed to the polar nature of the HA, thereby increasing the number of charge carriers in the copolyamide matrix [39]. Additionally, it is noteworthy that, even after the addition of a small percentage of filler, i.e., 1.5%, the dielectric constant of the COHA reached up to 6.5 from 3.2 for that of neat COPA at 100 Hz. Meanwhile, the dielectric constant of all the samples tested shows a decreasing trend, while increasing the frequency. This can be due to the fact that the interfacial dipoles fail to orient themselves along the applied field direction, resulting in a decrease in the interfacial polarization, eventually leading to a decreased dielectric constant [40].

#### 3.1.8. Contact Angle Analysis

**Figure 7:**
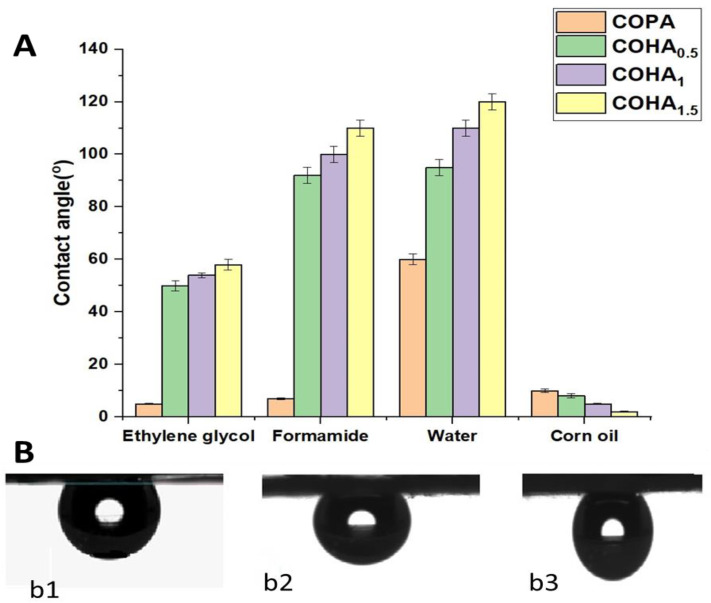
(**A**) Contact angle measurements for different solvents on neat COPA and COHA nanocomposite membranes. (**B**) Underwater oil contact angle (**b1**-COHA_0.5_, **b2**-COHA_1_ and **b3**-COHA_1.5_).

Figure 7A represents the contact angle measurements for various solvents on the neat COPA membrane and various composite COHA membranes. Due to the presence of –CO and –NH functional groups on its skeletal structure, electrospun COPA is generally classified as a hydrophilic polymer. It is to be noted that the contact angle of a liquid on a material surface depends on many factors, including the surface roughness, nature of material, morphology of the material, surrounding atmosphere, etc. As per the Cassie model, the surface roughness is inversely related to wettability, which means that as the surface roughness increases, the wettability decreases, thereby increasing the contact angle values [41]. The average contact angle of ethylene glycol, formamide, water and corn oil in air for neat COPA membrane is 5°, 7°, 60° and 10°, respectively. Meanwhile, adding the tubular nanoclay to the fiber causes variation of the surface properties, thereby affecting the contact angle values for different solvents. For COHA_1.5_, the average contact angle of ethylene glycol, formamide, water and corn oil in air is found to be 58°, 110°, 120° and 2°, respectively.

For real-time applications, such as oil/water separation, simple contact angle measurements in air would not give an exact idea on the behavior of membranes toward different liquids. From the measured values, low contact angle of oils with the membrane might create an impression that the membranes are not suitable for oil/water separation. However, mimicking the real conditions involved in oil/water separation, the underwater contact angle measurements of membranes for corn oil reveals entirely different results. The average underwater contact angle of corn oil on the COPA, COHA_0.5_, COHA_1_ and COHA_1.5_ were 128 ± 9 °, 131 ± 3°, 133 ± 2° and 136° ± 4° (Figure 7B). This implies that the clay composite nanofibre membrane followed the Cassie–Baxter model, i.e., good hydrophobic property with increased water contact angle [42]. These values highlight the underwater oleophobic nature of the composite membrane, probably due to the difference in surface properties caused by the presence of fillers. The porosity of the membranes was calculated and was found in the range 78.1–85.3%. Nanoclay composite membranes showed improved porosity, which also contributes to the higher oil/water emulsion separation capacity.

### 3.2. Oil/Water Emulsion Separation Experiment

**Figure 8:**
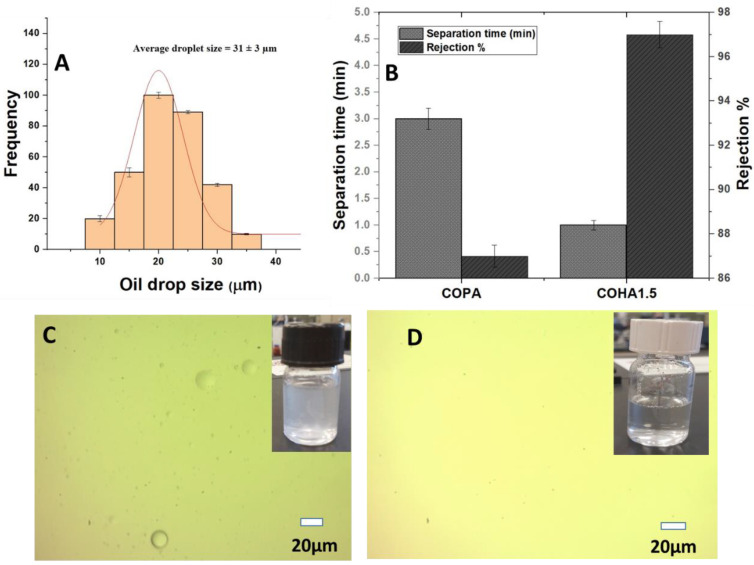
(**A**) Size distribution curve of oil/water emulsion. (**B**) Oil/water emulsion separation of copolyamide composite mats. (**C**) Optical microscopy image of 100 ppm emulsion before filtration. (**D**) Permeate of 100 ppm emulsion with inset digital image.

The as-prepared electrospun nanocomposite mats were tested for separation of oil from an oil/water emulsion. A total of 100 ppm vegetable oil in water was used for the study and the emulsion was filtered through the membrane under pressure. The filtrate was subjected to UV visible spectroscopic analysis and by comparing with the calibration curve, the ratio of oil present in the filtrate was deduced. Compared to the neat COPA membrane, COHA_1.5_ has a lower separation time that is almost more than two times faster with better oil rejection properties. It was found that the neat COPA electrospun mats were able to separate 87 wt% of oil, whereas the COHA_1.5_ composite electrospun fiber membrane showed improved oil retention capacity and removed 97 wt% of the oil (Figure 8A. The water permeation capability of the neat COPA membrane was found to be 2087 L/m^2^ h, whereas that of the COHA_1.5_ was 6265 L/m^2^ h, which is higher compared to the other halloysite-based composites reported [28,29]. The optical microscopy image of the 100 ppm oil/water emulsion and the permeate after filtration is shown in Figure 8C,D, respectively. It can be seen that after the filtration, the oil droplets in the water are not visible in the image, which indicates the removal of oil droplets. Hence, by controlling the surface roughness, the directional transport of water can be modulated and hence, more efficient emulsion separation can be achieved.

**Figure 9:**
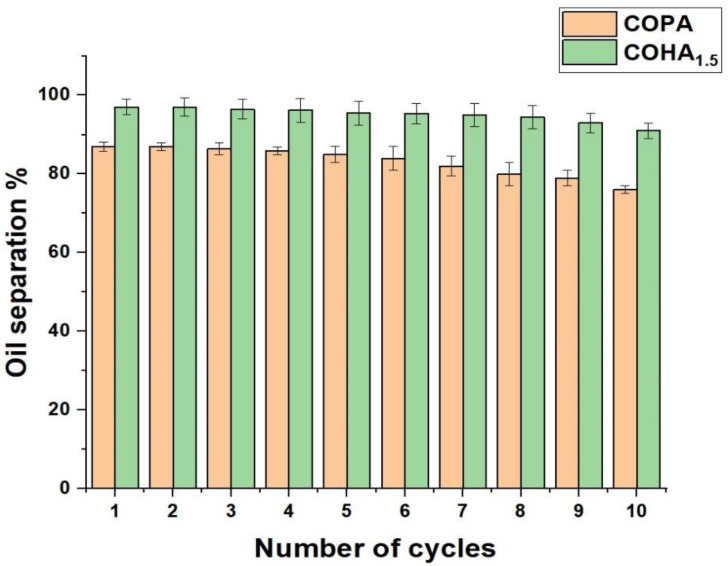
Reusability of the membrane for oil/water emulsion separation.

The reusability of the mats for oil/water emulsion separation was tested several times. Corn oil adsorbed on the mats were removed through extraction, using n-hexane. The oil/water separation test was performed 10 times, using the same electrospun membranes. Figure 9 shows that there was no drastic decrease in the efficiency of the membrane, even after reusing the membrane for several cycles.

## 4. Conclusions

In this study, we reported the preparation and characterization of electrospun polymer nanocomposite membranes based on a copolyamide matrix and halloysite nanoclay as the filler component, using a relatively green solvent n-propanol. Compared to other reported systems using halloysite clay for oil/water separation, this system is easy to fabricate, and no chemical modification of the filler is needed. Membranes with uniform fiber morphology and improved surface properties were obtained.

The major findings from this study include the following:

1. Electrospun polymer composite membranes with uniform fiber morphology were synthesized using copolyamide as the polymer matrix and halloysite clay nanotubes as inorganic nanofillers by the simple electrospinning technique, without any further chemical modifications of the components.
2. The addition of clay nanotubes improved the morphological, thermal, dielectric properties and liquid wettability properties of the polymer.
3. The contact angle values of COHA_1.5_ for ethylene glycol, formamide, water and corn oil in air were 58 °C,110 °C, 120 °C and 2 °C, respectively, while those of COPA were 5 °C, 7 °C, 60 °C and 10 °C. The underwater oil contact angle of COPA was 128 ± 9 °C, while that of COHA_1.5_ improved to 136 °C ± 4 °C.
4. The COHA_1.5_ showed an oil removal capacity of 97%, compared to the 87% by neat COPA. The water permeation capability of the COPA was 2087 L/m^2^ h, whereas COHA_1.5_ was found to be 6265 L/m^2^ h.
5. The as-synthesized COPA–halloysite clay composite electrospun membranes showed asymmetric wettability, which in turn, helped the directional liquid transport, thereby decreasing the separation time of water from the oil/water emulsion by up to three times faster, and the rejection performance of composite fiber was 10% higher than the neat copolyamide membrane.

## Figures and Tables

**Table 1:** Sample description and various parameters of electrospun copolyamide–HA composite nanofibers.

Sample Name	HA%	Viscosity of Solution (Pa × s)	Fiber Diameter (nm)	Sa (µm)	Porosity (%)	Average Pore Size (nm)
COPA	0	1.41	966 ± 200	3.4	78.1	701 ± 110
COHA _0.5_	0.5	1.79	1265 ± 152	4.5	81.2	756 ± 116
COHA_1_	1	1.81	1349 ± 250	5.8	83.5	810 ± 212
COHA_1.5_	1.5	1.84	1040 ± 145	6.6	85.3	840 ± 227

## Data Availability

The data presented in this study are contained within this article.
